# The serological diagnosis of adult coeliac disease – a cautious step forward? 

**Published:** 2018

**Authors:** Lauren JS Marks, Matthew Kurien, David S Sanders

**Affiliations:** *Academic Unit of Gastroenterology, Royal Hallamshire Hospital, Glossop Road, Sheffield, S10 2JF, UK *

 As described in a fascinating review by Holmes and Ciacci (1), the road to a ‘no biopsy’ serology-based diagnosis of adult coeliac disease (CD) makes perfect clinical sense. Who would want to have a gastroscopy if they can avoid it?! Furthermore, avoidance of gastroscopy and biopsy has the potential in the UK to be economical from the perspective of a cash-strapped NHS. There have been numerous studies supporting this approach (2,3). However, the positive predictive value (PPV) frequently cited of up to 100% for serological testing has its limitations (4-9). Many of the data sets are retrospective and from highly selected populations where the CD prevalence is high (6 studies, CD prevalence between 21-100%) (4-10). A unique endoscopy-based study, which has the advantage of 100% biopsy/histology being available, suggests less promising results. In this prospective study of 2000 unselected patients attending for endoscopy the CD prevalence was 3.9% (11). By contrast to the reported 100% PPV, this study demonstrates the PPV of tTG was only 28.6%, despite sensitivity and specificity of greater than 90%. This only increased to 71.7% when combined with a positive endomysial antibody (EMA) (11). We appreciate that this is a historical study using a recombinant human tissue transglutaminase (tTG) linked to gliadin-specific peptides. Nevertheless, it illustrates the variation that may occur in low coeliac prevalence populations. 

The ESPHGAN guidelines are to be commended for specifying that when recommending criteria for serology only diagnosis, they apply only to children and adolescents with signs and symptoms typical of CD (this should increase the prevalence of celiac disease in the population and therefore the PPV) (12). They then suggest selecting only paediatric cases that have a tTG x10 the upper limit of normal (ULN). In these patients the ESPGHAN guidelines suggest referral to a paediatric gastroenterologist, with testing of both EMA and Human Leukocyte Antigen (HLA) typing (‘triple strategy’) (12). Only then the diagnosis of CD can be made by an expert, with a gluten free diet (GFD) commenced and avoidance of a gastroscopy and biopsy under a general anaesthetic. Recently, the same investigators have demonstrated that HLA testing does not add value to the diagnosis and may not be required in future suggested strategies (13).

How would this approach translate to adult practice? There are a number of caveats yet to be satisfied: first, it should be noted that most patients diagnosed in adult practice do not have classical presentations of CD with malabsorption, and so arguably would not be eligible for serology only diagnosis under the ESPGHAN guidelines. Second, there are many reasons for undertaking a gastroscopy and duodenal biopsies in adults with suspected CD, which do not apply to paediatric practice but make common sense in adult practice (Table 1). Third, the reliance on very positive tTG also depends on the accuracy and comparability of test kits and labs in determining tTG levels, which may not happen even in reference laboratories (14-16).

**Table 1.** Reasons for proceeding with a biopsy in suspected adult coeliac disease

**1**	Antibodies are not 100% positive predictive
**2**	Patients may take reassurance in having a histological diagnosis.
**3**	Patients with either Irritable Bowel Syndrome or Crohn’s disease of the small bowel can be *pseudo- *improved by having a gluten free diet.
**4**	Baseline histology can allow assessment of severity (degree of villous atrophy) and give the patient confidence about an improvement of histology, if future biopsies are taken.
**5**	Some centres will not prescribe a gluten free diet unless the diagnosis of CD is proven.
**6**	A diagnosis of CD has implications for family members as up to 10% of first degree relatives are affected.
**7**	Many patients need an upper gastrointestinal endoscopy anyway as they have anaemia or other significant symptoms.
**8**	A gastroscopy is more easily tolerated by adults and does not require a general anaesthetic.
**9**	Occurrence of seronegative CD in some patients would result in missed diagnoses

An important consideration is the occurrence of villous atrophy in the presence of negative coeliac serology in adult patients. Although rare, seronegative CD is one of the most common causes of unexplained villous atrophy, and biopsies are essential in these patients in order to make a diagnosis (17). There are numerous causes of non-coeliac villous atrophy, such as small bowel bacterial overgrowth, Giardiasis, Whipple’s disease, mycobacterium tuberculosis and HIV enteropathy, all of which need to be ruled out via investigations carried out at gastroscopy prior to a diagnosis of seronegative CD (17). Biopsy avoidance would have catastrophic consequences for these patients, with an abundance of diagnoses being missed. Many clinicians currently ensure patient review and even repeat biopsy after treatment on a gluten free diet if the initial diagnostic biopsy is equivocal.

The greatest concern of all may be the ‘law of unforeseen consequences’! Serology is widely available and predominantly performed in primary care.^ 16^What could unfold is a ‘trial of GFD’ in primary care for patients with positive antibodies. Recently, Italian investigators have shown that when a diagnosis of CD has been made in ‘real’ clinical practice without undertaking a duodenal biopsy the diagnosis was incorrect in two thirds of these cases (18). 

In contrast, there is evidence to suggest that overreliance on serological markers may lead to underdiagnosis of CD. A North American study found that 2.4% of the 9665 patients referred for gastroscopy over a 30-year period displayed histological changes of CD on biopsy – a substantially higher prevalence than that reported from studies relying solely on serological screening (19).

For this reason, we would suggest that it is just as important to exclude CD as it is to make a positive diagnosis. We have recently described that 13% of the population report symptoms as a consequence of consuming gluten and that 2.9% of the general population are on a GFD even though they do not have coeliac disease (20). 

To advance our practice and cement a no biopsy policy into adult practice we require a multi-centre prospective study which will provide: 1) high quality data on the percentage of adult patients that do not require a biopsy; 2) The PPV and validity of numerous serological titres; 3) The role of HLA in the pathway for adults with suspected coeliac disease. Finally and crucially we require a clear message to all clinicians that positive serology equates to immediate referral to a Gastroenterology Consultant and not a presumptive diagnosis of CD and trial of a GFD in primary care! The road has been cleared by our paediatric colleagues. Now we must provide the evidence for adult practice to follow suit. 

## Conflict of interests

The authors declare that they have no conflict of interest.
